# Assessment of Pharmacy Information System Performance in Selected Hospitals in Isfahan City During 2011

**Published:** 2013-02-13

**Authors:** Sakineh Saqaeian Nejad Isfahani, Razieh Mirzaeian, Mahbobe Habibi

**Affiliations:** 1 School of Medical Records Teaching, Isfahan University of Medical Sciences, Isfahan, IR Iran

**Keywords:** Evaluation, Clinical Pharmacy Information Systems, Pharmacy

## Abstract

**Background:**

In supporting a therapeutic approach and medication therapy management, pharmacy information system acts as one of the central pillars of information system. This ensures that medication therapy is being supported and evaluated with an optimal level of safety and quality similar to other treatments and services.

**Objectives:**

This research aims to evaluate the performance of pharmacy information system in three types of teaching, private and social affiliated hospitals.

**Patients and Methods:**

The present study is an applied, descriptive and analytical study which was conducted on the pharmacy information system in use in the selected hospitals. The research population included all the users of pharmacy information systems in the selected hospitals. The research sample is the same as the research population. Researchers collected data using a self-designed checklist developed following the guidelines of the American Society of Health-System Pharmacists, Australia pharmaceutical Society and Therapeutic guidelines of the Drug Commission of the German Medical Association. The checklist validity was assessed by research supervisors and pharmacy information system pharmacists and users.

To collect data besides observation, the questionnaires were distributed among pharmacy information system pharmacists and users. Finally, the analysis of the data was performed using the SPSS software.

**Results:**

Pharmacy information system was found to be semi-automated in 16 hospitals and automated in 3 ones. Regarding the standards in the guidelines issued by the Society of Pharmacists, the highest rank in observing the input standards belonged to the Social Services associated hospitals with a mean score of 32.75. While teaching hospitals gained the highest score both in processing standards with a mean score of 29.15 and output standards with a mean score of 43.95, and the private hospitals had the lowest mean scores of 23.32, 17.78, 24.25 in input, process and output standards respectively.

**Conclusions:**

Based on the findings, the studied hospitals had minimal compliance with the input, output and processing standards related to the pharmacy information system. It is suggested that the establishment of a team composed of operational managers, computer fields experts, health information managers, pharmacists as well as physicians may contribute to the promotion of the capabilities of pharmacy information system to be able to focus on health care practitioners' and users' requirements.

## 1. Background

Nowadays health system managers regard the performance of pharmacy as the determining parameter in health system achievement to carry patients effectively (1). Hospital pharmacy department plays an active role in providing health care services by covering all areas of medicine use including selection, preparation, distribution, prescription and administration of medication and finally monitoring of patient outcomes (2). Each beneficiary in the medication supply chain requires a reliable and standard system. This system must be able to define who may have access to what information and how these information can be used, validated and managed (3). Health information system can be considered as a useful strategy for increasing the quality of health care system (4). Providing the information that physicians need so as to evaluate, respond and match such information with the medical processes (5), promotes the safe care and finally results in the availability of patient's information throughout the treatment course (6). Hospital Pharmacy Information System (PIS) as one of the main applications of information technology plays an increasingly essential role in meeting the efficiency, effectiveness, service quality and finally the patients' level of satisfaction (7). PIS was Established in early 1980s as an evolutionary phenomenon in the health care industry which was intended to provide medication services to promote patients' safety (8). PIS as a systematic system, investigates and validates all the policies related to the medication use process (9), provides patients, pharmacists, physicians, nurses and other health care providers with accurate, comprehensive and complete medication information to meet medication care needs (10). American Society of Health-System Pharmacists (ASHP) connotes that pharmacists play an increasingly significant role in ensuring the positive results of medication therapy (11). It decreases the errors due to unreadable transcriptions, drug administration and delivery, furthermore PIS makes the practitioners and professionals aware of the unsafe coefficient of the medications, overdose prescriptions, potential interacting effects of prescribing two medications simultaneously (12). PIS Data bank related to the administered medications plan commonly includes patients' demographic information, medications' medical-pharmacological classification, bar coding of the drugs, generic and brand names of the drugs, dosage strength and route of administration, medications' producer company, distribution date and quantity (13). Using the PIS, the prescribers can review the deferred orders and omit the unnecessary orders, hence, enlightening the patients and medication providers (14). Wager (2005) has enumerated a number of PIS capabilities in his study including clinical screening to manage medication interactions, dosage control of the medications considering the age, weight and other effective factors, managing the drug orders to track all prescriptions, inventory management by maintaining a proper list of inventory, and bar coding of drugs to control current and past prescribed drugs and patients' physiological parameters, to name but a few (15). The PIS as an integrated information system has a significant effect on the reduction of drug errors especially drugs preventable side effects (9). Despite this, based on Health Care Management and Regulatory Information Company (HCPro) estimation, medication errors contribute to 30.5% of the total fatal medical errors (16) .Furthermore, according to the National Patient Safety Agency's (NPSA) report, the total number of medication errors had been 991 in a period of 5 year from 2000 to 2004 with the effective dosage error as the most frequent kind (26.9%). In addition, with respect to the total number of errors associated to the various dimensions of dosage, the most common was belonged to overdose (50.9%) (17). PIS plays a critical role in preventing drug misuses (18). One of the practical seven aspects of PIS relating to the time prior to using the medications is resorting to the 7 rights comprising. "right patient, right dose, right route, right time, right drug, right information and finally right documentations" (2, 19). According to the previous research, informational components including drug information, patients' information and prescribers' information are recorded in the PIS applied in the Shahid Beheshti University's associated medical and teaching hospitals (50.1%, 21.9% and 33.3%, respectively). Furthermore, the functions of PIS are processed in 39.2% of the hospitals and reported in 66.8% of them (20). In one study, Martin (2006) showed that applying the technology of automated dispensing systems, using smart infusion pumps, recording medications information clinically, bar-coding of drugs, recording physicians' orders in computers, preparing electronic medical profiles had promoted the productivity of the organization under study up to 65%, 53%, 29%, 29%, 27% and 43%, respectively (12). Mohammed Al-Soltan in his study entitled " Hospital pharmacy practice in Saudi Arabia: Prescribing and Transcribing in the Riyadh region" in 2011 found that 51.9% of the total hospitals were equipped with the electronic system for recording the medications (21). Regarding the role of advanced technology as the highest parameter to justify the preference of electronic health system over the manual pharmacy activities (22, 23) on the one hand, and the capabilities of the PIS to improve the quality of the services related to the medication production, dispensing, maintenance and monitoring and the significance of the information for the efficient and effective management of the pharmacy on the other, this research has embarked upon the evaluation of this system based on the American, German and Australian Societies of Health system Pharmacists standards, by taking into account the informational components including input, processing and output components.

## 2. Objectives

This research aimed to evaluate the performance of pharmacy information system in three types of teaching, private and social affiliated hospitals.

## 3. Patients and Methods

This study is applied, descriptive and analytical and cross-sectional in nature. The research population included all users of pharmacy information system used in the hospitals in Isfahan city. This system has been used in 10 teaching hospitals (i.e. Shahid Beheshti, Shahid Chamran, Noor, Ali Asqar, Imam Musa Kazem, Isa Ibn Maryam, Al-zahra, Ayatollah Kashani, Feiz, Seyed Al-Shohada and Amin hospitals). Although 7 private hospitals (Khanevadeh Clinic, Sa'adi, Sina, Sepahan, Isfahan Clinic, Mehregan, Hazrat -e- Zahra-e- Marzieh) and 2 hospitals associated to Social Services (i.e. Shariati and Qarzi) were found to use the system. The research sample is the same as the research population. Researchers used a self-designed checklist to collect data, containing 236 informational components, which have been designed according to the informational guidelines issued by the Societies of Health-System Pharmacists in America, Germany and Australia based on three informational standards, including input, processing and output. The American Societies of Health-System Pharmacists (ASHP), Pharmaceutical Society of Australia (PSA) and therapeutic guideline of the Drug Commission of the German Medical Association (DCGMA) have compiled some guidelines and announcements to focus on the role of pharmacists, medication consultation and the importance of information in medication therapy process. The designed checklist has benefited from these guidelines in three areas of input, processing and output. The validity of the compiled checklist was assessed based on the results of conducted reviews and the supervisor and advisor professors' points of view conducting the present research as well as the computer sciences field experts and the professors in the health information management field and finally, the pharmacists.

The researchers collected the required data through observation and the questionnaires were distributed among PIS pharmacists and users in hospitals in question. After final control, the collected data was entered the SPSS18 software, Statistical Package for the Social Sciences (SPSS), for further analysis. Data was analyzed using descriptive statistics including frequency and partial frequency. The researchers applied Kruskal Wallis and Wilcoxon nonparametric tests trying to investigate to what extent both the type of the hospital (teaching, private and social services) and the type of PIS applied accords with the standards related to the input, processing and output components. Thereafter, the situation of the selected hospitals was analyzed and compared accordingly.

## 4. Results

Among the hospitals in question (10 teaching, 7 private and 2 social services hospitals), the PIS was of semi-automated type in 84.21% of the hospitals and of automated type in 15.79%. Totally, the medication inventory information system used in these hospitals was found to be automated in 63.16% of the hospitals and manual in 36.84%.

Table 1 shows the comparison of the mean scores related to the level of compliance with the informational components of input, output and processing standards among the hospitals under study. Kruskal-Wallis test indicated that there was no statistically significant difference among the hospitals for meeting the input standards in their Pharmacy Information System (P = 0.17 & ƛ_2_= 3.46). However, the processing and output standards were found to be statistically different with the significance level of 0.01 (P = 0.03 and ƛ_2_ = 6.70). To conduct the pair comparison of the hospitals regarding processing and output standards, Wilcoxon test was used. This test results showed that there was statistically significant difference between teaching (P = 0.01, Z = -2.39) and private hospitals (P = 0.01, Z = -2.49) with a significance level of 0.05.

**Table 1:** The Comparison of Mean Scores Related to the Degree of Meeting Input, Processing and Output Standards According to the Society of Pharmacists Guidelines in the Piss Applied in the Selected Hospitals in Isfahan City

	Teaching	Private	Social Services
**Input standards**			
Mean score	29.68	23.32	32.75
Standard deviation	8.94	6.53	0.48
**Processing standards**			
Mean score	26.15	17.78	21
Standard deviation	6.79	4.99	1.41
**Output standards**			
Mean score	43.95	24.25	41.56
** Standard deviation**	10.86	12.79	5.34

The level of observing the input, processing and output standards given by the Society of Health-System Pharmacists were also investigated for the type of PIS applied in the hospitals in question. The comparison of the mean scores has been presented in Table 2. Based on Kruskal-Wallis test results, there was no statistically significant difference between the applied PISs for the degree of meeting the input (P = 0.62, ƛ_2_ = 5.28), processing (P = 0.58, ƛ_2_ = 5.60) and output standards (P = 0.72, ƛ_2_ = 4.44).

**Table 2:** The Comparison of Mean Scores Related to the Degree of Compliance to the Input, Processing and Output Standards According to the Society of Pharmacists for the Local Software Information System Applied

Local Softwares	Sayan Rayan Ekbatan	Kowsar (old version)	Kowsar (new ersion)	Pouya Samaneh	Lohgostar	Rayavaran Touseeh	Modiriyate Amar	Social services	Total
**No.**	6	2	1	2	4	1	1	2	19
**Input standards**									
**Mean score**	25.57	30.86	25.51	32.27	25.43	21.37	29.82	32.75	27.66
**Standard deviation**	11.49	11.21	0	8.53	6.09	0	0	0.48	8.17
**Processing standards**									
**Mean score**	27.41	22.25	19	19.25	19.12	16	27	21	22.52
**Standard deviation**	7.05	13.78	0	1.76	6.45	0	0	1.41	6.91
**Output standards**									
**Mean score**	38.27	41.27	39.53	35.46	30.23	18.60	47.09	41.56	36.44
**Standard deviation**	18.06	27.13	0	13.97	11.85	0	0	5.34	14.39

**Figure 1:**
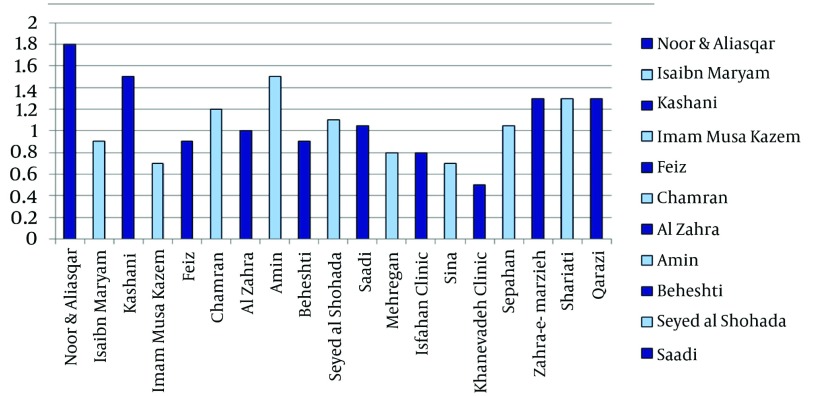
The Comparison of the Mean Scores for Meeting the Input, Processing and Output Standards in the Piss for the Teaching Hospitals

**Figure 2:**
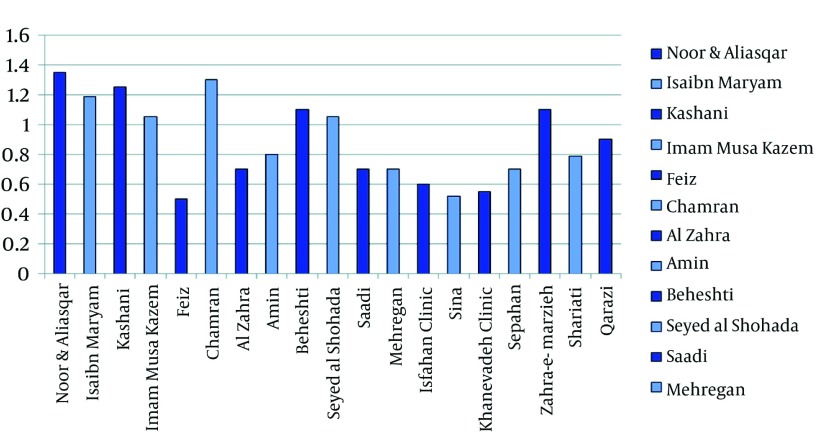
The Comparison of the Mean Scores for Meeting the Input, Processing and Output Standards in the Piss for the Private Hospitals

**Figure 3:**
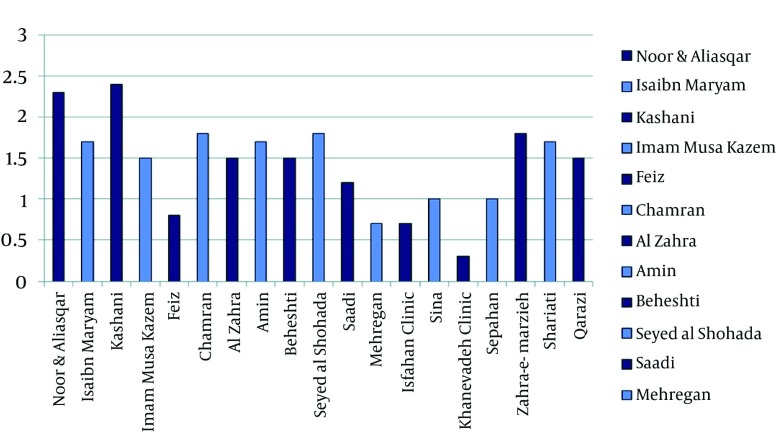
The Comparison of the Mean Scores for Meeting the Input, Processing and Output Standards in the Piss for the Social Services Hospitals

The results of the comparison of the mean scores related to the degree of observing the input, processing and output standards in the PISs for both the type of the hospital and local softwares of information system applied have been summarized in Figures 1, 2 and 3. Among the teaching hospitals, the software of Pharmacy Information System applied in Noor and Ali Asqar hospitals took the first place in observing the input with a mean score of 45.68% and processing standard with a mean score of 34%, while the software (old version) in Kashani hospital with a mean score of 58.72%, enjoyed the highest rank as far as output standards were concerned. The software PISs applied in the Imam Musa Kazem took the last place with a mean score of 18.96% for input standards. Furthermore, the least mean scores in both observing processing standards (12.50%) and output standards (22.09%) found to belong to software (old version) applied in the Feiz hospital. Among the private hospitals under study, the software of PIS in Kaneveadeh Clinic and Sina hospital took the last place in observing input with a mean score of 12.93% & output with a mean score of 4.65% standards and processing (12.50%) standards, respectively.

## 5. Discussion

Based on the study findings, it appears that the hospitals in question with a maximum mean score of 14.50 are very far from the desirable condition regarding the input standards. The input standards itself is composed of a number of informational components namely registration of information related to the medication (including usage patterns, pharmacodynamics and pharmacokinetics properties, drug allergies and drug interactions, dosage, and other factors), access to patients' demographic information, access to patients' claims information, signs and progress course of the illness, patients' treatment information, records of drug prescriber's information, records of the purchase control information and drug inventory, information about observing the standards, instructions and guidelines on documentation and exchange of information in the PIS.

Currently the administrative managers' informational requirements play a critical role in whether executing a PIS will be successful, the requirements which would help them in future decisions. Hence, appropriate attention must be paid to these requirements. The results of the present study are in consistence with Ursula's study titled "Pharmacy services to England's Emergency Departments in 2010". This study revealed that just 40% of the medication information requirements are recorded in the Pharmacy Information Systems in form of description. This provides some evidence that the potential functions of system in supporting the management of medication-related complications and order writing skills on the one hand and decreasing drug interactions and managing medication inventory on the other, have been ignored (24). Likewise, Azizi (2005) in his study found that the mean scores of meeting the standards determined by the American Physicians College in order writing & medication information maintenance in the PISs applied in Iran, Tehran and Shahid Beheshti Universities of Medical Sciences were 28.5%, 26.6% and 31.1%, respectively (25). With respect to observing processing standards, the hospitals are very far from the desirable condition (i.e. 100) with a maximum mean score of 12.95.

Although the accurate calculation of the dosage is one of the primary elements of medication therapy to reach the desirable result (9), so that if properly calculated, it will prevent drug interactions and complications related to the medication therapy. Unfortunately, PIS applications scope in the selected hospitals was just limited to the calculation of the costs and quantity of the medications dispensed to patients. PIS Significant potential capacities becomes clear when we review the results of John Nazzaro's study on the pharmacies equipped with computerized systems in Naval and Charleston hospitals (1983) during 2 years. This study illuminated that providing the opportunity to calculate the medication dosage for the out patients, computerized systems have led to the promotion of productivity of the pharmacy with an 18% increase in work load, and a 14% decrease in staffing (26). In another study entitled "Pharmacist Workload and Pharmacy Characteristics Associated With the Dispensing of Potentially Clinically Important Drug-Drug Interactions" performed in the state of Arizona in 2007, Mallon et al. (2007) reached findings similar to those of the present research. Investigating the pharmacies in question from different perspectives including workload, use of technology in prescription, processing drugs interactions warns and the viewpoints of the pharmacists regarding the medication interactions by using software, showed that most of the pharmacies (81.1%) under study were part of pharmacy chain organizations (27).

Regarding the output standards, with a maximum mean score of 12.90, the hospitals in question are again far from the optimal condition (100). PIS should also be used for creating different reports related to the pharmacy performance including:

a) Daily reports related to dispensing the medications under control based on dispensing location.

b) Reports related to the required medications to be purchased.

c) Reports related to medication inventory.

d) Reports related to medication prices.

e) Reports related to annual performance of the pharmacy.

f) Reports related to the financial status of the pharmacy.

g) Reports related to the medications inventory at the end of the year (9).

PIS also plays an increasingly large role in providing both the physicians and other care providers or patients at the time of his or her discharge with a report of medication therapy he or she has received which would be useful for continuing the treatment course. Among the researches conducted in this regard, Paul's study titled "Impact of Pharmacist-Facilitated Hospital Discharge Program 2009" is worth noting. According to this study, all patients after being discharged have experienced some difficulties regarding the medications they need after discharge. These complications mainly are related to the quantity or the type of required medications resulting from several medication changes during stay time. For the treatment and control group under study, the rate of medication contradictions at the time of discharge found to be 33.5% and 59.6%, respectively (28). Hence, regarding the prolific research performed on the modern technologies and taking into account the role of PIS in health care domain, this fact becomes clear that, this system must be regarded as a clinical system too, rather than merely a technical system.

PIS plays a key role in decreasing the errors, increasing the speed and facilitating the processes from three discrete perspectives which are as follows:

I. Managing the optimal medication services (covering medication supply, maintenance and distribution)

II. Optimal financial management (including costs, profits and investments)

III. Scientific support to medication therapy (including calculating

Medication dosage accurately, preventing drugs potential interactions, predicting drug-allergies and controlling side effects of the prescribed drugs).

Contrary to this, the findings of the present research revealed that in the hospitals in question the use of PIS as a component of Hospital Information System (HIS) was just restricted to the managerial and financial aspects of medication services processes without having any role in the medications related scientific or usage dimensions.

As a result, PIS's advantage in reducing medication errors remains untouched.

It can also be inferred from the results analysis that PIS applied in the selected hospitals have failed not only to satisfy their key role in promoting the treatment process and decreasing the medication errors but also quite contrary to the expectations, each has had varied performance (e.g. see results related to Sayan Rayan Ekbatan software and Kowsar software (old version)). This condition can be attributed to inattention to the users' needs and what they expect from such systems and not giving them an opportunity to take part in administering this system on the one hand, and ignoring the role of pharmacists' clinical consultation beneficial in patients treatment.

Keeping these results in mind, it can be claimed that as an inevitable requirement of health care system, Iran's health system authorities must undertake to administer an integrated PIS throughout the country.

Pharmacy Information System is composed of three data banks, including patient information, medication information (managerial, financial and scientific data) and medication prescriber information. It is expected that this system could be effective in three fields of operational management, financial management and scientific support to the medication services in hospitals. In addition, it is also expected that administering and using these data banks lead to errors reduction and increase the speed of managing the orders and dispensing the medications. Hence, when PIS is to be administered, informational requirements and hardware, software, manpower and educational resources needed for establishing the medications plans, standards, policies and laws must be taken into account.

In sum, the findings of the present research showed that among the 8 local software information systems in question, the Social Services System and Pouya Samaneh software gained the highest ranks in observing the input standards, while the highest mean score in meeting the standards related to processing and output standards belonged to Sayan Rayan–e-Ekbatan software and Modiriat-e-Amar software, respectively.

Due to inattention to such system's capabilities, all of them suffer from some deficiencies which need to be obviated.

Since one of the most significant and effective elements of PIS, i.e. medication's scientific data bank has been ignored and the PISs in question have not been equipped with some software suitable for providing the prescriber with scientific backup in his or her making decision process, the prescribers have just relied on their own information making medication errors in health care domain inevitable.

### 5. 1. Suggestions

Before designing and administering PIS, enough attention must be paid to the users' informational requirements as well as their expectations from the system. The availability of informational elements effective on managing and monitoring the medication related complications, reducing medication errors, checking the medication therapy information to ensure the suitability of medication regimen and medication warns to identify drug allergies, system potential relation with other systems supporting physicians in their making decisions and at last, recording the prescribers' orders are some capabilities which can promote this system so that it can be regarded as a clinical system with a therapeutic approach.
